# Supplementary material for: Genome-Wide Association Study Identifies a Rice Panicle Blast Resistance Gene, Pb2, Encoding NLR Protein
Source: Int J Mol Sci. 2022 May 18;23(10):5668. doi: 10.3390/ijms23105668 (PMC9145240; doi:10.3390/ijms23105668)
Supplement: Supplementary file 1 [file ijms-23-05668-s001.zip › ijms-1708389-supplementary/Supplementary Materials/Figures S1-S5 and Table S1.pdf]

A

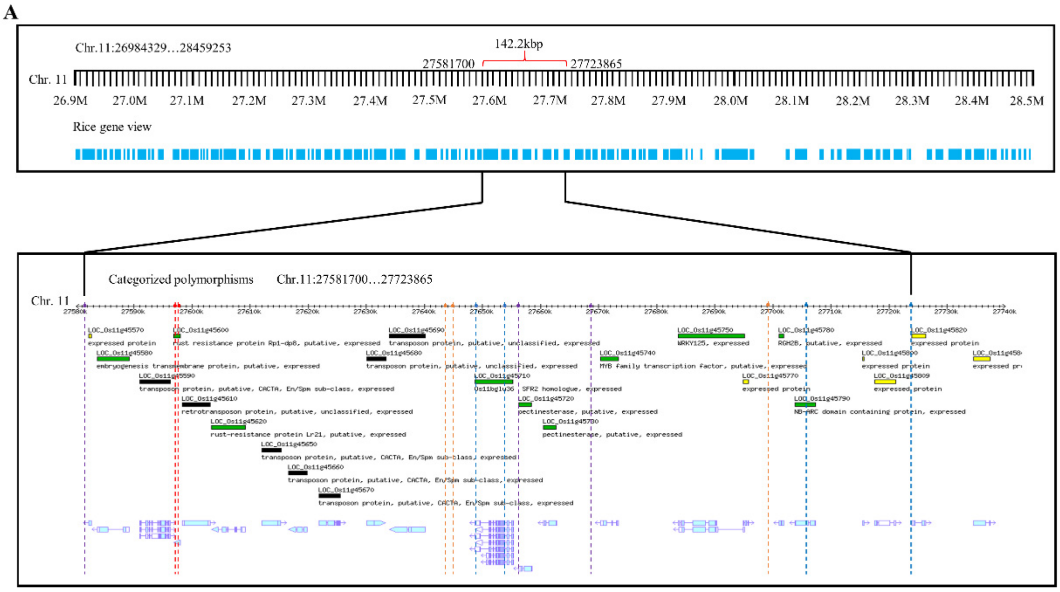

B

Group I (2 polymorphism in 1 gene) (red)

Chr.	Position (bp)	P-value	Ref	Alt	Gene ID	Region	Ref.codon	Alt.codon	Ref.aa	Alt.aa	Annotation
11	27597071	6.73	T	C	LOC_Os11g45600	CDS	CAT	CGT	H	R	rust resistance protein Rp1-dp8, putative, expressed
11	27597338	4.32	T	C	LOC_Os11g45600	CDS	AAG	AGG	K	R	rust resistance protein Rp1-dp8, putative, expressed

Group II (4 polymorphisms in 5 genes) (purple)

Chr.	Position (bp)	P-value	Ref	Alt	Gene ID	Region	Ref.codon	Alt.codon	Ref.aa	Alt.aa	Annotation
11	27581700	4.03	C	T	LOC_Os11g45570	promoter	—	—	—	—	expressed protein
11	27581700	4.03	C	T	LOC_Os11g45580	promoter	—	—	—	—	embryogenesis transmembrane protein, putative, expressed
11	27655356	7.4	G	T	LOC_Os11g45720	promoter	—	—	—	—	pectinesterase, putative, expressed
11	27668861	6.1	C	T	LOC_Os11g45740	Promoter	—	—	—	—	MYB family transcription factor, putative, expressed
11	27723865	7.43	C	G	LOC_Os11g45820	5′ UTR					expressed

Group III (3 polymorphisms in 2 genes) (blue)

Chr.	Position (bp)	P-value	Ref	Alt	Gene ID	Region	Ref.codon	Alt.codon	Ref.aa	Alt.aa	Annotation
11	27648994	7.22	T	C	LOC_Os11g45710	3′ UTR	—	—	—	—	Os11bglu36 SFR2 homologue, expressed
11	27653919	7.21	C	T	LOC_Os11g45710	INTRON	—	—	—	—	Os11bglu36 SFR2 homologue, expressed
11	27705881	4.27	T	A	LOC_Os11g45790	CDS	GCA	GCT	A	A	NB-ARC domain containing protein, expressed

Group IV (2 polymorphisms) (orange)

Chr.	Position (bp)	P-value	Ref	Alt
11	27643836	6.26	A	G
11	27644922	5.39	G	A
11	27699196	4.73	C	T

Group V (217 polymorphisms)

Figure S1. DNA polymorphisms in the candidate regions of the panicle blast resistance peak on chromosomes. (A) The distribution of significant SNP(-Log10P≥4) of the candidate interval (27581700-27723865bp) on chromosome 11. Groups classified as I-IV are indicated by red, purple, blue and yellow dots, respectively. Transposons and retrotransposons are represented by black rectangles, expressed proteins are represented by yellow rectangles, and functional proteins are represented by cyan rectangles. (B) Detailed information on the DNA polymorphisms in the candidate region.

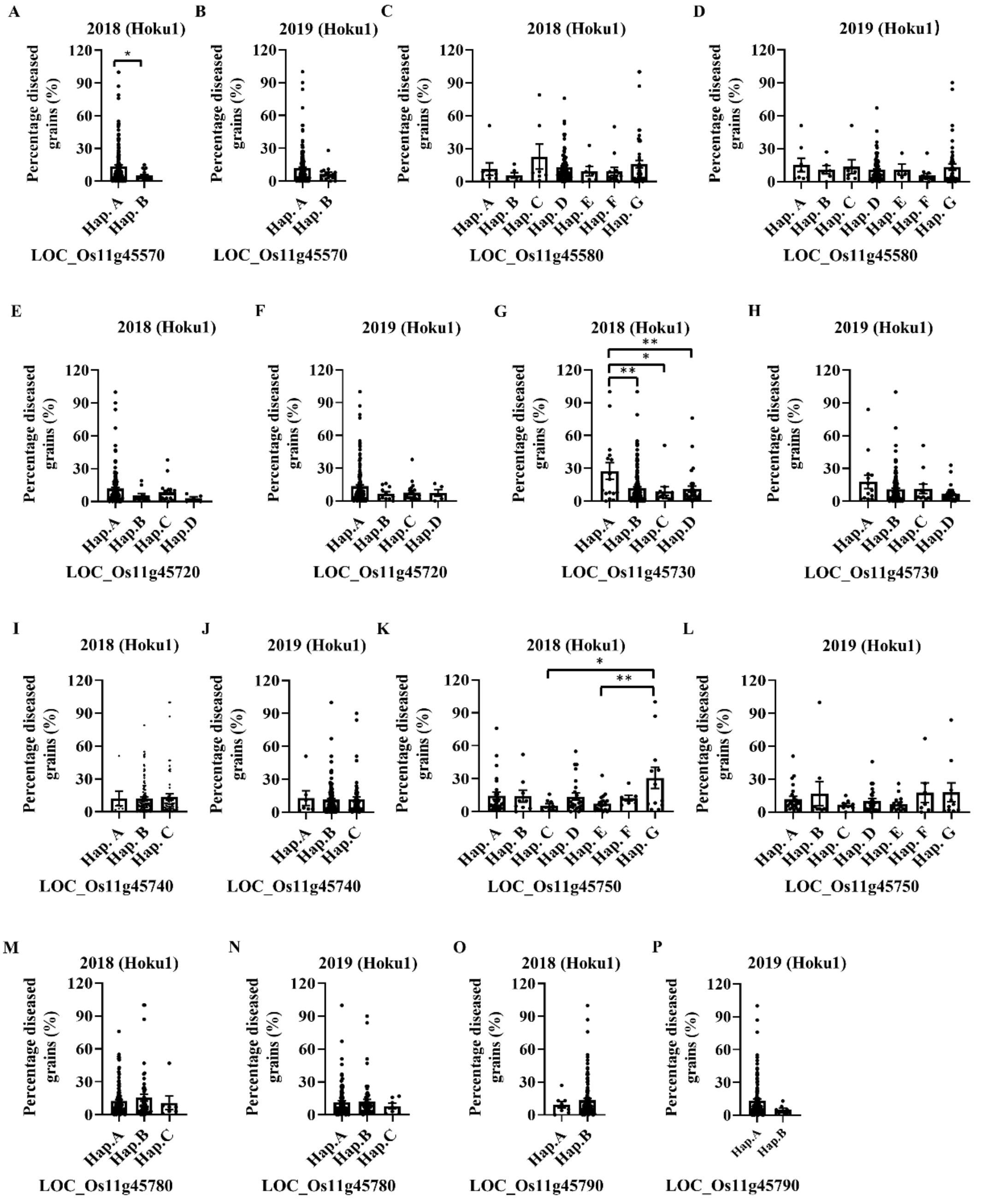

Figure S2. Haplotype of the SNP polymorphism candidate region. (A-P) Percentage diseased grains (%) caused by different haplotypes. Presented as a scatter plot with bars, the edge of the box represents the mean ± SEM, and each point represents the percentage diseased grains for a germplasm. Differences between haplotypes were tested with GaphPad Prism 8.3 t test, *P<0.05, **P<0.01.

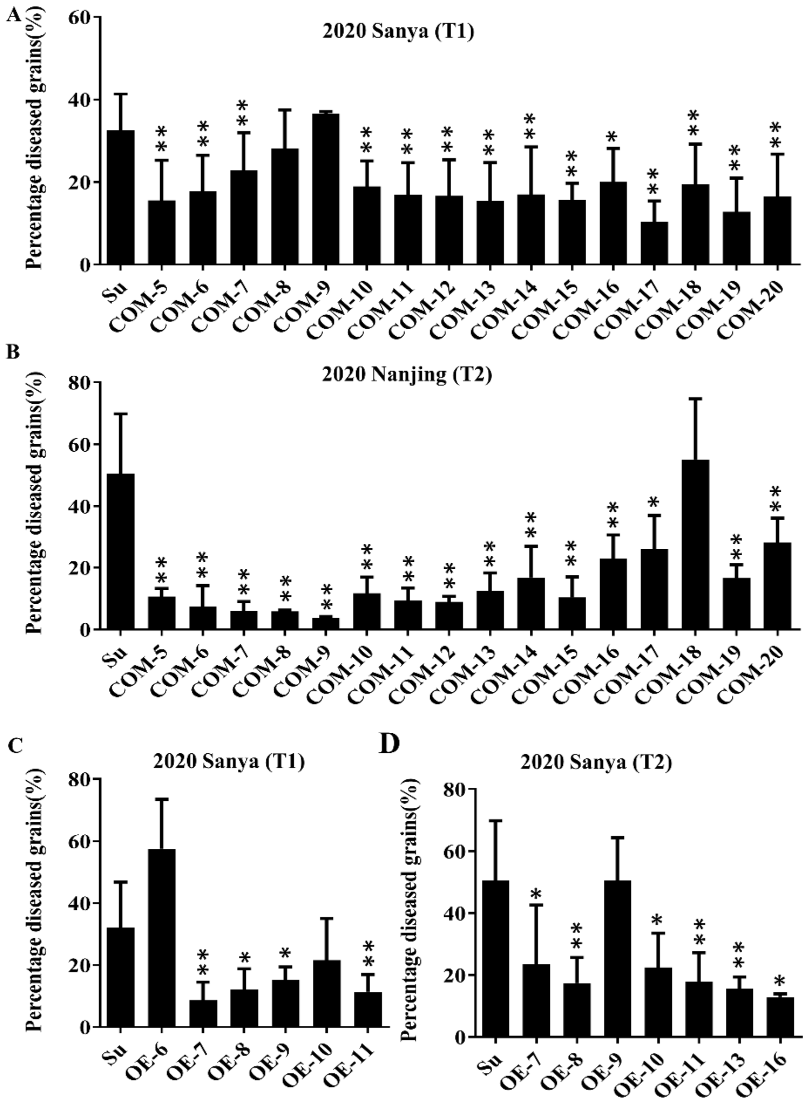

Figure S3. Complementation and overexpression of *Pb2* in susceptible materials can reduce the percentage diseased grains. (A-D) Percentage diseased grains (%) of WT and transgenic plants. The percentage diseased grains was counted three weeks after inoculation, and each bar represents the average of 9 panicles. Error bars represent standard deviation, and differences between WT and transgenic plants were tested with GaphPad Prism 8.3 t-test, *P<0.05, **P<0.01.

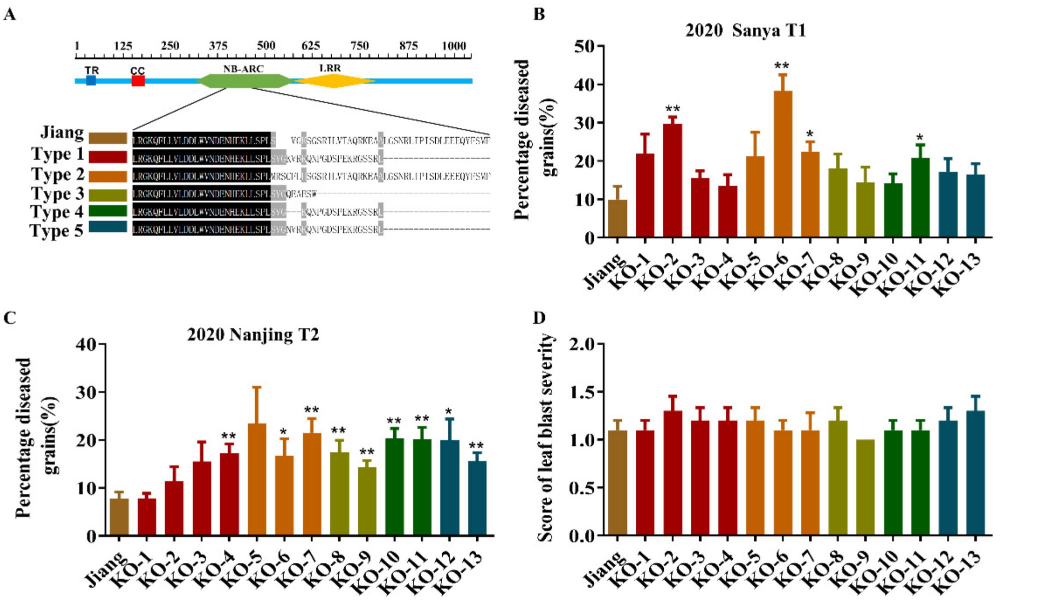

Figure S4. Resistance changes after *Pb2* knockout are not significant. (A) gRNA target location and mutation type. TR: transmembrane domain. CC: coiled-coil. NB-ARC: resistance protein nucleotide binding region. LRR: leucine-rich repeat. Type 1, Type 3, Type 4 and Type 5 translation termination, and Type 2 had four amino acid substitutions and three amino acid insertions in the NB-ARC domain. (B,C) Percentage diseased grains (%) of WT and CRISPR knockout mutants. The percentage diseased grains was counted three weeks after inoculation, and each bar represents the average of 9 panicles. Different colors are used to distinguish mutation types. Error bars represent standard deviation, and differences between WT and transgenic plants were tested with GaphPad Prism 8.3 t-test, *$P<0.05$, **$P<0.01$. (D) Assessment of blast resistance in CRISPR knockout mutants. After the sixth day of inoculation, only brown necrotic spots were found in all materials without obvious expansion.

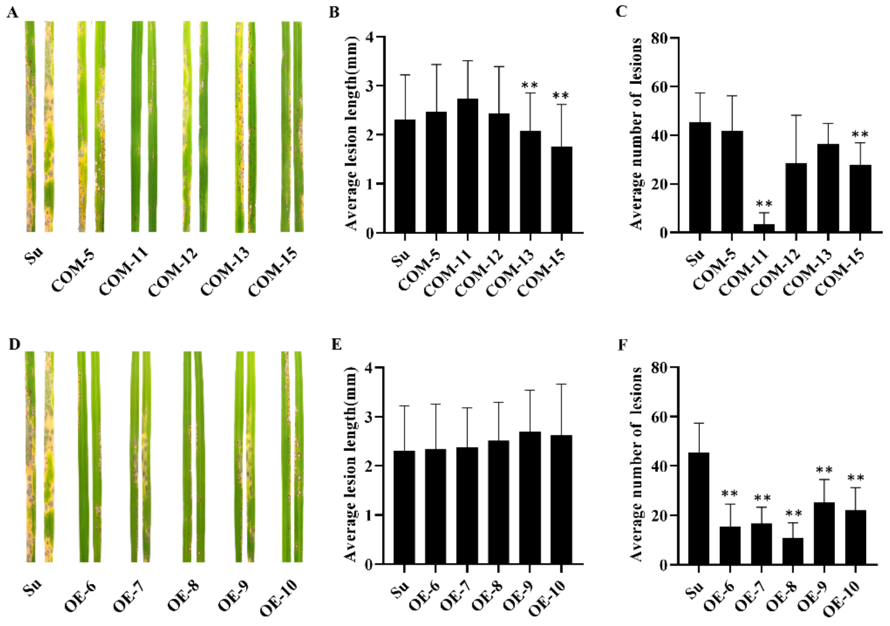

Figure S5. Complementation and overexpression of *Pb2* can reduce the number of lesions. (A,D) Response of three-week-old seedlings 6 days after inoculation with *Hoku1*. (B,C,E,F) Average lesions length and average number of lesions in WT and transgenic plants. Lesion length and number were measured 6 days after inoculation. Each bar is the mean of at least 20 seedlings. Error bars represent standard deviation, and differences between WT and transgenic plants were tested with GaphPad Prism 8.3 t-test, *$P<0.05$, **$P<0.01$.

Table S1. Candidate genes information screened based on SNP location.

Locus [1]	Chr.	Gene ID [2]	SNP location [3]	Gene annotation [4]
PBRL-10/18	11	LOC_Os11g45570	CDs	expressed protein
PBRL-10/18	11	LOC_Os11g45580	CDs	expressed protein
PBRL-10/18	11	LOC_Os11g45590	Promoter	transposon protein, putative, CACTA, En/Spm sub-class, expressed
PBRL-10/18	11	LOC_Os11g45600	CDS	rust resistance protein Rp1-dp8, LRR, putative, expressed
PBRL-10/18	11	LOC_Os11g45610	No SNP [5]	retrotransposon protein, putative, unclassified, expressed
PBRL-10/18	11	LOC_Os11g45620	CDS	rust-resistance protein Lr21, NB-ARC, putative, expressed
PBRL-10/18	11	LOC_Os11g45650	Promoter	transposon protein, putative, CACTA, En/Spm sub-class, expressed
PBRL-10/18	11	LOC_Os11g45660	No SNP	transposon protein, putative, CACTA, En/Spm sub-class, expressed
PBRL-10/18	11	LOC_Os11g45670	No SNP	transposon protein, putative, CACTA, En/Spm sub-class, expressed
PBRL-10/18	11	LOC_Os11g45680	No SNP	transposon protein, putative, unclassified, expressed
PBRL-10/18	11	LOC_Os11g45690	No SNP	transposon protein, putative, unclassified, expressed
PBRL-10/18	11	LOC_Os11g45710	CDs	Os11bglu36 SFR2 homologue, Galactosyltransferase
PBRL-10/18	11	LOC_Os11g45720	CDs	pectinesterase, putative, expressed
PBRL-10/18	11	LOC_Os11g45730	CDs	pectinesterase, putative, expressed
PBRL-10/18	11	LOC_Os11g45740	CDs	MYB family transcription factor, putative, expressed
PBRL-10/18	11	LOC_Os11g45750	CDs	WRKY125, expressed
PBRL-10/18	11	LOC_Os11g45770	Promoter	expressed protein
PBRL-10/18	11	LOC_Os11g45780	CDs	RGH2B, NB-ARC, putative, expressed
PBRL-10/18	11	LOC_Os11g45790	CDs	CC-NBS-LRR domain containing protein, expressed
PBRL-10/18	11	LOC_Os11g45800	Promoter	expressed protein
PBRL-10/18	11	LOC_Os11g45809	Promoter	expressed protein
PBRL-10/18	11	LOC_Os11g45820	5′ UTR	expressed protein

[1] The identified panicle blast resistance-related locus (PBRL-10/PBRL-18) was repeated at the end of the long arm of chromosome 11.

[2] All gene IDs predicted within the candidate interval (27581700-27723865) based on the MSU version 7.0.
[3] The location of the SNP (including significant and non-significant) in the candidate interval (27581700-27723865) on the gene.
[4] Gene function prediction was performed by the Rice Genome Annotation Project (http://rice.uga.edu/).
[5] No SNP falls on this gene.
